# Enterprise Food Fraud in China: Key Factors Identification From Social Co-governance Perspective

**DOI:** 10.3389/fpubh.2021.752112

**Published:** 2021-11-19

**Authors:** Liangyun Niu, Mo Chen, Xiujuan Chen, Linhai Wu, Fu-Sheng Tsai

**Affiliations:** ^1^School of Economics, Anyang Normal University, Anyang, China; ^2^School of Economics and Management, Nanjing University of Aeronautics and Astronautics, Nanjing, China; ^3^School of Business, Institute for Food Safety Risk Management, Jiangnan University, Wuxi, China; ^4^Department of Business Administration, Cheng Shiu University, Kaohsiung, Taiwan; ^5^Center for Environmental Toxin and Emerging-Contaminant Research, Cheng Shiu University, Kaohsiung, Taiwan; ^6^Super Micro Mass Research and Technology Center, Cheng Shiu University, Kaohsiung, Taiwan

**Keywords:** food fraud, business ethics, social co-governance, safety and quality, DEMATEL-based ANP

## Abstract

Food fraud not only exacerbates human public health risks but also threatens the business development of food and related industries. Therefore, how to curb food fraud effectively becomes a crucial issue for governments, industries, and consumers. Previous studies have demonstrated that enterprise food fraud is subject to joint influences of factor at various hierarchical levels within a complex system of stakeholders. To address enterprise food fraud, it is necessary to identify the key such factors and elucidate the functional mechanisms, as well as systematic analysis of the interrelationships among clusters and factors. Hence, we grounded on a social co-governance perspective and investigated the food fraud key influencing factors and their interrelationships in an emerging food market – China, by using the DEMATEL-based analytic network process (DANP). Results showed that the identified key cluster was government regulation, social governance, and detection techniques. Four other key factors were also identified, including government regulatory capability and penalty intensity, expected economic benefits, maturity of market reputation mechanism, and transparency of supply chain. Policy implications from the social co-governance perspective for China and similar economies are discussed finally.

## Introduction

Food fraud is a collective term used to encompass the deliberate substitution, addition, tampering, or misrepresentation of food, food ingredients, or food packaging or false/misleading statements made about a product for economic gain (1–5). With the escalation in incidents, scope, and harm, research on food fraud has increased in recent years (6, 7). In recent years, food fraud has become a serious and challenging issue for worldwide society (8–10). Food fraud grows obstacles for food safety regulation and the food industry (11, 12). It also increases human health risks (13), hinders development of the food market/industries, and causes trust issues among stakeholders, including food producers and dealers, consumers, trading partners, and regulatory authorities (14). Food fraud can also be more difficult to expose and can carry greater threat than conventional food safety issues (15).

For example, one of the most notorious food fraud cases worldwide was the discovery in 2008 of the illegal addition of melamine, an industrial raw material, to infant milk powder in China, which negatively impacted 300,000 infants, six of whom died (16, 17). The company in question is a large enterprise group with a history of over 50 years and total assets of nearly RMB 2 billion (as of the end of 2007). In order to reduce costs, the enterprise involved used water and melamine in the milk to counterfeit. This is an example of food fraud as 'commercial enterprise crime' carried out by producers in the food supply chain. The discovery in 2013 of the addition of horsemeat to certain products in many European countries is another example of significant food fraud (18). Such incidents have led governments and relevant organizations in various countries to step up food safety regulation (19). For instance, In China, the government has repeatedly restructured its food safety regulatory bodies, reformed regulatory rules and practices, and promulgated the highly stringent *Law on Food Safety* protocols, which are targeted at effective regulation (20, 21).

Previous food safety regulations, however, are not designed to curb deliberate misconduct and are therefore not effective at addressing intentional food fraud (22, 23). Existing systems (and research on them) only focused on the compliance of food producers with food safety control systems, particularly the *Hazard Analysis Critical Control Point* (HACCP) system, to minimize the microbial, chemical, and physical risks incurred during food production (24, 25). Food fraud is a deliberate behavior of a food producer (generally) and will include attempts to evade supervision and regulation (11). Lord et al. (26) emphasizes that food fraud constitutes a crime and generally occurs along the supply chain of ordinary food, similar to other criminal activities.

Existing food fraud research is heavily weighted toward food science, packaging and labeling, and legal areas of knowledge discovery (27). Enterprise food fraud is a business behavior performed under certain conditions. Moving forward, this requires a business decision-making perspective to further study the problem of food fraud in food companies (13). So far, Van Ruth et al. (23), Levi et al. (28), Meerza et al. (29–31) and other studies have initially discussed how various factors affect the fraud behavior of food companies. We have a clearer understanding of the fraudulent decision-making behavior of food companies and laid the foundation. For instance, Meerza et al. (31) studied the Optimal Policy Response to Food Fraud and found that under different circumstances, strict monitoring and enforcement and increased certification costs will have different effects on companies' food fraud behaviors. However, the existing research only analyzes the influence of various factors on the fraud behavior of food companies. So, among these factors, which ones are the key factors? What are their interrelationships between factors? Existing research ignores these important issues. According to our knowledge, there is currently no literature report that identifies the key factors that affect the food fraud behavior of companies and analyzes the internal relationships between the factors.

In addition, enterprise food fraud is not only influenced by the action of certain individual factors, but also joint, organizational actions of a complicated system of clusters (cluster is a factor sets formed by different factors of the same class) at different hierarchical levels and among factors within a cluster (32). However, studies on the correlations among clusters and factors that motivate enterprise food fraud and how such clusters and factors jointly influence enterprise food fraud remain limited.

Heeding to such thoughts of the gaps in the literature, we argue that studies need to explore key factors of food fraud from more systematic and holistic theoretical lens and methodology, such as the social co-governance perspective and the DEMATEL-based analytic network process (DANP) method proposed here. Social co-governance theory for food safety emphasizes on “social participation for the collective pursuit of food safety” (33). As compared with traditional governance approaches for food issues, social co-governance stresses more on the wide and collective efforts from a diverse set of stakeholders, which ensures better informational transparency/symmetry, risk and cost sharing capacity, and resource richness (34). Such relationship also stresses social contract beyond economic ones (35). For methodological concern, Huang et al. (36) and Wu et al. (37) found the DANP to be an effective approach for studying the correlations among factors and (more usefully) the inter- and intra-clusters (factor sets) relationships at different hierarchical levels.

From the analysis of the development history of food fraud, the problem of food counterfeiting exists in any country in the world with varying degrees. And food fraud often occurs in the highly competitive food market. China has a highly competitive and relatively mature food market, which is similar to the food markets in the United States and the European Union. However, about 50% of food safety incidents in China are caused by food fraud, which is the result of the combination of complex factors such as the huge return from food consumption market, the large number of food enterprises with insufficient integrity, and the weakness of food supervision in China, etc., which might be different from the United States and the European Union but similar to most developing countries. Therefore, using China as the research object to study food fraud is reasonably significant and is of positive value in understanding the causes of food fraud/counterfeiting in similar economies' contexts with potential measures that might be taken by the whole society.

## Literature Review

### Extant Literature

#### Production View

Information asymmetry between producers and consumers creates an adverse choice for oversupply of low-quality, unsafe products (38, 39). Traditional food regulation is dominated by the command-control type of intervention. In most developed countries, food safety regulation has focused on the imposition of standards that specify how food products should be produced and/or their final safety level (40). However, since the 1990s, food operators have frequently been given more responsibility to monitor food safety (41). For example, the UK Food Safety Act (1990) encourages food companies to establish private food safety control measures to ensure the quality and safety of food produced and sold (42). The EU Food Hygiene Regulations, implemented on January 1, 2006, require all food producers and operators to have food safety control measures to prove that they are managing food safety in their businesses. In United States, food safety control measures, such as HACCP, become a category of food safety regulation (43).

Food risks may be caused by malpractice of suppliers who exploit the fact that their production processes and resulting product properties cannot be directly observed by buyers (44). However, current food safety regulations aim to deal with unintentional food safety incidents such as microorganisms, physics and chemistry, rather than deliberately deceiving people. Besides, the design of food safety control measures from a production view could not take into account deliberate fraud. So, malicious intent is the blind spot of current food safety law (2). Food fraud often occurs outside the authorized supply chain and usually involves the addition of unsupervised substances (13). The fraudsters can design and manufacture adulterated materials based on the nature of the adulterated product, thereby escaping existing food safety controls (45). Therefore, existing food safety control measures are not effective against food fraud (22, 23).

#### Criminology View

Food fraud is crime-committed by producers and operators in the food supply chain to make full use of the opportunities of crime (26). In order to successfully implement food fraud, fraudsters actively seek for the opportunity and actively avoid detection using their technical expertise (46). In terms of the nature of food fraud, the collapse of the security of the entire food supply chain depends on a single factor, the criminal (45). Since food fraud is caused by conscious intelligent human opponents, food fraud is a crime and the crime prevention related theories have been applied in research (3, 4, 47, 48). The routine activity theory sees crime as the outcome of the convergence in time and place of (1) motivated offenders and (2) suitable targets in (3) the absence of capable guardians (23). Food fraud and other types of corporate crime have similar characteristics. In accordance with the routine activity theory, it is necessary to study the factors affecting the food fraud vulnerability from three aspects: opportunities, motivations and control measures, and develop a food fraud vulnerability assessment tool (23). From the view point of the Criminology, food fraud vulnerability assessment tools should be used, identify potential weaknesses of food systems, and to effectively prevent food fraud (49, 50). Nonetheless, if (as the present study does) the food fraud is defined as a commercial enterprise crime, then we need to extend such a crime prevention theory to designing organizational and institutional prevention strategies to enhance the integrity of the food system (26).

### Social –Co-governance Perspective

Based on the reviews above, we found that the production viewpoint does not take into account the deliberate characteristics of food fraud, so it is ineffective to control food fraud. Although the criminological viewpoint makes up for the above shortcomings of production viewpoint, and places its emphases on preventing food fraud through prevention. However, under the background of rapid development of food production technology and increasingly internationalization and complexity of food supply chain, it is far from enough to rely solely on the strength and resources of enterprises and governments to prevent food fraud. With the purpose to reduce costs and improve the effectiveness of food safety regulation, the new collaborations between public authorities and food operators in monitoring food safety has been developed (34, 40, 51). But, establishing a better food economy with sustainable development needs the efforts of all stakeholders and the integration of relevant resources. Involvement of all stakeholders to work together helps to improve the practicability of decision-making and reduce the burden on participants (33). Therefore, food safety risk governance must introduce the participation of consumers, non-governmental organizations and other social forces to guide the whole society to co-govern together (52).

In theory, social co-governance is rooted in the theory of cooperative governance. In the late 20th century, the role orientation of “super nanny” of the government in western countries' welfare systems resulted in many disadvantages, such as expansion of functions, overstaffed institutions and inefficiency, which caused public discontent due to inadequate governance of environmental protection, market monopoly, food safety and other issues (33). In order to solve the problems of fragmentation and decentralization of government governance, the theory of social governance, which emphasizes the multi-dispersed subjects to reach a multilateral interactive cooperative network, began to emerge at the end of the 20th century (53). As an important stakeholder of food safety, the media, employees, consumers and other social entities can also play an important role in preventing food safety risks. Social co-governance of food safety is a concept aimed at strengthening the partnership among the government, enterprises and social entities. The concept of social co-governance has become a practice in many countries. In the EU, governments, enterprises, social organizations, and citizens are actively involved in food safety governance. In China, the Food Safety Law of the People's Republic of China on October 1, 2015 established social co-governance as an important criterion for food safety risk governance. According to this, both theory and practice require that the discussion of the governance of food fraud be extended to the main constituents of a society.

In sum, the governance of food fraud with production and criminological views is mainly from the two main entities of the enterprise and the government, respectively. This paper expands the research scope to a pluralistic social groups (i.e., enterprise, government, consumers, and other stakeholders) based on the theory of social co-governance. A major reason is that the social co-governance theory suggests that stakeholders such as the consumers, social organizations or the other relatively neglected actors can also play an important role in ensuring food safety and in preventing fraud, posing a powerful complement to government governance and corporate self-discipline (33). To this end, based on our review of the perspective of social co-governance, this paper proposes five dimensions and 12 factors that may affect the corporate food fraud behavior (Table 1).

**Table 1:** Key clusters and factors influencing enterprise food fraud.

**Target**	**Cluster**	**Factor**
Enterprise food fraud	Enterprise characteristics (D_1_)	Enterprise scale (C_11_)
		Enterprise business ethics (C_12_)
		Manager's awareness of social responsibility (C_13_)
	Economic benefits and technical hardness of food fraud (D_2_)	Expected economic benefits (C_21_)
		Technical hardness of food fraud (C_22_)
	Government regulation, social governance, and detection techniques (D_3_)	Government regulatory capability and penalty intensity (C_31_)
		Supervision by social forces (C_32_)
		Utility of detection techniques and methodologies (C_33_)
	Market governance (D_4_)	Maturity of market reputation mechanism (C_41_)
		Consumption behavior on food market (C_42_)
	Internal relationship and transparency of food supply chain (D_5_)	Constraints by downstream enterprises (C_51_)
		Transparency of supply chain (C_52_)

#### Enterprise Characteristics Cluster

Enterprise food fraud is closely related to business scale, business ethics, and awareness of social responsibilities.

##### Enterprise Scale

Enterprise scale refers to the number of employees and the size of assets. Though an enterprise may engage in food fraud regardless of its scale (47), a food enterprise of smaller scale has higher risk of deliberate crime as it may choose not to recall sold products suspected of authenticity or safety problems and may ignore consumer grievances (54). For instance, Wu et al. (55) reported that small-scale enterprises are more inclined to abuse food additives. Levi et al. (28) also revealed that smaller farms are more vulnerable to risks and may resort to food fraud when facing quality uncertainty or price pressure.

##### Business Ethics

Business ethics refers to the integrity and ethical atmosphere within the enterprise. Business ethics are basic ethical codes that an enterprise complies with in all production and trade activities (56). Food fraud is unethical conduct (57), which is often closely related to business culture and the decision-maker's failure to stand behind the ethical bottom line (58). Business ethics is an important risk factor for corporate financial fraud (59, 60). Similarly, business ethics are key cultural factors leading to food fraud vulnerability (23). Enhancing an enterprise's business ethics imposes a positive influence on the enterprise from a cultural perspective and encourages the business to refrain from food fraud (61).

##### Manager's Awareness of Social Responsibility

Manager awareness refers to the attitude of managers toward the social responsibility that the enterprise should take. Social responsibilities are fundamental duties related to environmental protection, justice, and equality that an enterprise assumes while striving for maximum benefits (62). Furthermore, ensuring food safety is the most important social responsibility of a food enterprise (63). Though most enterprises understand social responsibilities of food safety for the sake of a good public image (64), the concept of social responsibility originated and evolved to promote compliance with ethics and legislation among increasing cases of non-compliance (65). Illegal conduct will decrease if an enterprise strictly adheres to its social responsibilities. A manager's awareness of these social responsibilities also influences both willingness and performance. The stronger the manager's awareness of social responsibilities, the more responsible an enterprise is in regard to food safety and food fraud misconduct (66).

#### Expected Economic Benefits and Technical Hardness Cluster

Food fraud is intrinsically subject to expected economic benefits and technical hardness.

##### Expected Economic Benefits

Although food fraud may require an input of resources, it will also undoubtedly generate benefits (67). This is why fraudsters choose to misbehave in violation of social ethics and even with the risk of punishment (68, 69). For example, Levi et al. (28) found that enterprise food fraud aims to maximize the perceived quality of low-quality products to achieve higher economic benefits. Bitzios et al. (70) also determined that foods bearing geographical indication (GI) labels usually resulted in better quality foods and higher consumer acknowledgment; furthermore, when substantial economic benefits are expected from counterfeiting ordinary food into a GI product, the enterprise exhibits a higher probability of committing food fraud.

##### Technical Hardness

An enterprise will be more inclined to commit fraud when it is technically easy (71). The technical hardness of fraud can be measured from both knowledge and substance aspects. On the knowledge side, a fraudster is usually a technical expert with rich knowledge of production and knows how to perform the fraud and how to evade capture (46). Furthermore, it is relatively easy to acquire the knowledge and techniques necessary for food fraud (23). On the substance side, most food fraud does not necessitate complicated equipment or other substances and the required additives are often easily available (72). For example, the infant milk powder incident in China resulted from adding melamine to milk to conceal that it had been diluted with water (16). Melamine is an ordinary and easily accessible chemical and its addition to the milk did not require any complicated techniques.

#### Government Regulation, Social Governance, and Detection Techniques Cluster

Food fraud is subject to impact from government regulatory capability and penalty intensity, social supervision, and utility of detection techniques and methodologies.

##### Government Regulatory Capability and Penalty Intensity

Food fraud rampancy is closely related to inefficient government regulation (73). In China, the deficiency in food safety supervision and control, as well as the fragmentation of regulatory agencies, has resulted in poor regulation (47) as well as increased opportunities for fraudulent behavior. In addition, the government sampling inspection system is based on conventional empirical methodologies, information, and knowledge (i.e., what additives may be supplemented to food), and often cannot identify fraud based on the latest developments (74, 75). Furthermore, there is no punitive “joint examination” on similar enterprises. Therefore, the food quality sampling inspection system itself does not effectively deter food fraudsters (76). In addition, relatively moderate punishment coupled with high expected economic benefits does not constitute an effective deterrent, thus resulting in the high risk of food fraud (54).

##### Supervision of Social Forces

Media, consumers, employees, and social organizations can help alleviate food fraud (33). For example, Peng et al. (77) found that food safety scandals disclosed by the media can lead to a decline in sales and damage to the brand's reputation, which might reversely help correct the misconducts of food producers. However, companies may be under the expectation that food fraud will not be discovered, which can induce enterprise misconduct. Traditionally, China is a institution-driven market economy with limited participation by civil society (78). If the general public identify and report fraud cases, those committing the fraud are exposed (69) and the enterprise manager's psychological expectations may change. The “whistler” inside the enterprise can help discover the fraudulent behavior (79). Waterhouse et al. (80) indicated that employees are more aware of hidden fraud and therefore whistle-blowing is a powerful tool to prevent fraudulent activities from inside food enterprises. Li et al. (81) stated that social organizations can help to avoid the dual failure of public government power and private market power and play an irreplaceable role in supervising the operation of food enterprises.

##### Utility of Detection Techniques and Methodologies

A fundamental reason why food fraud is rampant is the poor utility of food testing methodologies, which are unable to detect food fraud (11). Generally speaking, food testing methodologies are based on known additives and pollutants and whether such additives are excessive compared with the prescribed threshold values (82). However, the sophistication of food and raw materials complicates both analysis and detection (67), particularly when the testing institutes do not know the additives (6). Thus, in response to enterprise food fraud, it is important to combine targeted and non-targeted testing methods (19).

#### Market Governance Cluster

Food fraud is also subject to influence from the maturity of the market reputation mechanism and consumption behavior on the food market.

##### Maturity of Market Reputation Mechanism

Good market reputation can enhance market sales (83) and can be the primary means by which an enterprise avoids market risks and achieves economic benefits (84). Therefore, market reputation constitutes a foundation of survival and benefits. Food fraud can result in severe damage to an enterprise's market reputation (85), not just for the enterprise committing the wrongdoing, but also for other enterprises in the same industry, causing heavy economic losses. For example, the 2008 melamine infant milk powder incident in China damaged the reputation of the company involved so badly that it went bankrupt in the same year. Therefore, reputation is a key market mechanism for preventing enterprise food fraud (54). For instance, a mature market reputation mechanism, whereby any enterprise food fraud is disclosed to the general public, can deter other enterprises from committing such misconduct.

##### Consumption Behavior on Food Market

Regulation of food systems exists to ensure safety and enhance consumer confidence in the food which they purchase and consume. However, food fraud scandals have caused consumers to be anxious and distrustful of local food products, and further stimulate distrust in food system. Consumers' awareness of food fraud incidents has reduced consumers' willingness to pay for products from companies and industries that have experienced food fraud scandals (86). Moreover, when consumers believe that there is a lack of regulatory protection, they will develop strategies to reduce the risk of food fraud to prevent the purchase and consumption of fraudulent food (54). The three main coping approaches include purchasing decision making, information searching & sharing and daily self-preservation strategies (87). These risk mitigation strategies of consumers (that is, consumer behavior) affect the food fraud behavior of companies.

#### Internal Relationship and Transparency Along Food Supply Chain Cluster

Mutual constraints among stakeholders and transparency along the food supply chain are also key factors influencing enterprise food fraud.

##### Constraints by Downstream Enterprises in the Supply Chain

Previous studies have demonstrated that downstream enterprises in the supply chain can constrain upstream enterprises by inspecting the safety and authenticity of foods or materials, thus preventing food fraud. Babich and Tang (88) and Cao et al. (89) showed that inspection and deferred payment mechanisms can prevent adulteration by suppliers and upstream enterprises. Nevertheless, deficiencies in the constraints mechanism by downstream enterprises can also increase the probability of enterprise food fraud. Levi et al. (28) revealed that, compared with concentrated supply chains, distributed supply chains entail difficulties for downstream enterprises to impose constraints on upstream enterprises, thus raising the probability of food fraud along the supply chain.

##### Transparency of Supply Chain

Increasing complexity of the supply chain network can result in less visibility of the operational management of suppliers and is a key cause of food fraud (70). For example, Waterhouse et al. (80) determined that adulterated wine can reach consumers due to the non-transparent chain of supply and distribution. The melamine infant milk powder scandal in China also provides evidence that non-transparency of the upstream supply chain can lead to food fraud (61). Ensuring transparency of the supply chain can enhance food safety and quality (90). The Safe Supply of Affordable Food Everywhere (91) organization states that efforts should be made to acquire and maintain enhanced traceability information to ensure high transparency of the supply chain and minimization of food fraud.

## Methods and Data

### Methods

Based on the existing literature, this paper has summarized some factors and clusters that influence the counterfeiting decisions of food companies, so what is the interrelationship between clusters and factors? What are the intrinsic mechanisms upon which they can influence food counterfeiting? What are the key clusters and key factors? To answer the questions, based on Hsu et al. (92) and Huang et al. (36), we applied the DANP method as follows:

#### Acquired the Influential Net Relationship Map With DEMATEL

Step 1 – Calculated direct relationship average matrix *A*. Firstly, a direct relationship matrix was generated based on the assessment results of each expert member. The average matrix *A* = [_*a*_*ij*_]*n*×*n*_, *i, j* = 1, 2, …, *n* was then obtained by calculating the average of the same factor of all direct relationship matrices.
Step 2 – Calculated initial direct influence matrix *D*.
  $$\begin{array}{l} D=z\times A\\ z=\min\left\{1/\max_{i}\overset{n}{\underset{j=1}{\sum }}a_{ij},1/max_{j}\overset{n}{\underset{i=1}{\sum }}a_{ij}\right\},\\ \text{where}i,j\in \left\{1,2,\ldots ,n\right\} \end{array}$$
Step 3 – Calculated total influence matrix *T*.
  $$T={\left[t_{ij}\right]}_{n\times n},i,j=1,2,\ldots ,n$$
  where, *t*_*ij*_ is the degree of the direct and indirect influences of factor *i* on factor *j*.
  $$\begin{array}{l} T=D+D^{2}+D^{3}+\ldots +D^{h}=D\left(I-D^{h}\right){\left(I-D\right)}^{-1}\\ \text{As }D={\left[d_{ij}\right]}_{n\times n},0\leq d_{ij}<1,0\leq \sum_{i}d_{ij}\leq 1,0\leq \sum_{j}d_{ij}\leq 1,\\ \text{when }h\to \infty ,D^{h}={\left[0\right]}_{n\times n}, \end{array}$$
  then
  $$\begin{array}{l} T=D{\left(I-D\right)}^{-1} \end{array}$$
Step 4 – Calculated the sum of each line and each column of total influence matrix *T*.
  $$\begin{array}{lll} r_{i} & = & \overset{n}{\underset{j=1}{\sum }}t_{ij}\\ c_{j} & = & \overset{n}{\underset{i=1}{\sum }}t_{ij} \end{array}$$
  where, *r*_*i*_ is the total of the direct and indirect influences of factor *i* on other factors in the system and *c*_*j*_ is the total of the direct and indirect influences that factor *j* receives from other factors in the system. When *i* = *j*, *r*_*i*_ + *c*_*i*_ is the sum of influence that factor *i* imposes on other factors and receives from other factors and *r*_*i*_ − *c*_*i*_ is the difference of influence that factor *i* imposes on other factors and receives from other factors. *r*_*i*_ − *c*_*i*_ > 0 indicates that factor *i* has influence on other factors and is the cause factor in the system. *r*_*i*_ − *c*_*i*_ < 0 indicates that factor *i* is influenced by other factors and is the result factor in the system.
Step 5 – Acquired the influential net relationship map.

#### Calculated the Mixed Weight by Combining DEMATEL and the Analytic Network Process

Assuming each cluster has an equal degree of influence, ANP standardizes an unweighted supermatrix established by pair comparison between indicators into a weighted supermatrix. However, different clusters have different influences on enterprise food fraud. Therefore, DEMATEL can be used to determine the degree of influence of each cluster and thus normalize the ANP unweighted supermatrix to simulate real-world situations (92).

Step 1 – Acquired the unweighted supermatrix. We first divided the total influence matrix T into the *T*_*D*_ matrix (by cluster) and *T*_*C*_ matrix (by factor) based on clusters and factors in Table 1.
  $$\begin{array}{l} T_{C}=\begin{array}{c} D_{1}{\begin{array}{c} \vdots \\ c_{1m_{1}} \end{array}}^{c_{11}}\\ D_{i}{\begin{array}{c} \vdots \\ c_{im_{i}} \end{array}}^{c_{i1}}\\ D_{n}{\begin{array}{c} \vdots \\ c_{nm_{n}} \end{array}}^{c_{n1}} \end{array}\overset{\begin{array}{ccccc} \underset{C_{11\cdots C_{1m_{1}}}}{D_{1}}\; & \cdots & \underset{C_{j1\cdots C_{j}m_{j}}}{D_{i}}\; & \cdots & \underset{C_{n1\cdots C_{nm_{n}}}}{D_{n}}\; \end{array}}{\left[\begin{array}{ccccc} T_{C}^{11} & \cdots & T_{C}^{1j} & \cdots & T_{C}^{1n}\\ \vdots & & \vdots & & \vdots \\ T_{C}^{i1} & \cdots & T_{C}^{ij} & \cdots & T_{C}^{in}\\ \vdots & & \vdots & & \vdots \\ T_{C}^{n1} & \cdots & T_{C}^{nj} & \cdots & T_{C}^{nn} \end{array}\right]}\\ T_{D}=\left[\begin{array}{ccccc} t_{D}^{11} & \cdots & t_{D}^{1j} & \cdots & t_{D}^{1n}\\ \vdots & & \vdots & & \vdots \\ t_{D}^{i1} & \cdots & t_{D}^{ij} & \cdots & t_{D}^{in}\\ \vdots & & \vdots & & \vdots \\ t_{D}^{n1} & \cdots & t_{D}^{nj} & \cdots & t_{D}^{nn} \end{array}\right] \end{array}$$
  We then calculated the standardized total influence matrix $T_{c}^{a}$.
  $$\begin{array}{l} T_{C}^{\alpha }=\begin{array}{c} D_{1}{\begin{array}{c} \vdots \\ c_{1m_{1}} \end{array}}^{c_{11}}\\ D_{i}{\begin{array}{c} \vdots \\ c_{im_{i}} \end{array}}^{c_{i1}}\\ D_{n}{\begin{array}{c} \vdots \\ c_{nm_{n}} \end{array}}^{c_{n1}} \end{array}\overset{\begin{array}{ccccc} \underset{C_{11\cdots C_{1m_{1}}}}{D_{1}}\; & \cdots & \underset{C_{j1\cdots C_{j}m_{j}}}{D_{i}}\; & \cdots & \underset{C_{n1\cdots C_{nm_{n}}}}{D_{n}}\; \end{array}}{\left[\begin{array}{ccccc} T_{C}^{\alpha 11} & \cdots & T_{C}^{\alpha 1j} & \cdots & T_{C}^{\alpha 1n}\\ \vdots & & \vdots & & \vdots \\ T_{C}^{\alpha i1} & \cdots & T_{C}^{\alpha ij} & \cdots & T_{C}^{\alpha in}\\ \vdots & & \vdots & & \vdots \\ T_{C}^{\alpha n1} & \cdots & T_{C}^{\alpha nj} & \cdots & T_{C}^{\alpha nn} \end{array}\right]}\\ \text{where},T_{C}^{\alpha 11}=\left[\begin{array}{ccccc} t_{c11}^{11}/d_{c1}^{11} & \cdots & t_{c1j}^{11}/d_{c1}^{11} & \cdots & t_{c1m_{1}}^{11}/d_{c1}^{11}\\ \vdots & & \vdots & & \vdots \\ t_{ci1}^{11}/d_{ci}^{11} & \cdots & t_{cij}^{11}/d_{ci}^{11} & \cdots & t_{cim_{1}}^{11}/d_{ci}^{11}\\ \vdots & & \vdots & & \vdots \\ t_{cm_{1}1}^{11}/d_{c1}^{11} & \cdots & t_{cm_{1}j}^{11}/d_{cm_{1}}^{11} & \cdots & t_{cm_{1}m_{1}}^{11}/d_{cm_{1}}^{11} \end{array}\right],\\ d_{ci}^{11}=\sum_{j=1}^{m_{1}}t_{ij}^{11},i=1,2,\ldots m_{1}. \end{array}$$
  Finally, we calculated the unweighted supermatrix *W*.
  $$\begin{array}{l} W=\left(T_{C}^{\alpha }\right)\prime \end{array}$$
Step 2 – Calculated the weighted standardized supermatrix *W*^α^.
  $$\begin{array}{l} T_{D}^{\alpha }=\left[\begin{array}{ccccc} t_{D}^{11}/d_{1} & \cdots & t_{D}^{1j}/d_{1} & \cdots & t_{D}^{1n}/d_{1}\\ \vdots & \vdots & \vdots & \vdots & \vdots \\ t_{D}^{i1}/d_{i} & \cdots & t_{D}^{ij}/d_{i} & \cdots & t_{D}^{in}/d_{i}\\ \vdots & \vdots & \vdots & \vdots & \vdots \\ t_{D}^{n1}/d_{n} & \cdots & t_{D}^{nj}/d_{n} & \cdots & t_{D}^{nn}/d_{n} \end{array}\right]\\ =\left[\begin{array}{ccccc} t_{D}^{\alpha 11} & \cdots & t_{D}^{\alpha 1j} & \cdots & t_{D}^{\alpha 1n}\\ \vdots & \vdots & \vdots & \vdots & \vdots \\ t_{D}^{\alpha i1} & \cdots & t_{D}^{\alpha ij} & \cdots & t_{D}^{\alpha in}\\ \vdots & \vdots & \vdots & \vdots & \vdots \\ t_{D}^{\alpha n1} & \cdots & t_{D}^{\alpha nj} & \cdots & t_{D}^{\alpha nn} \end{array}\right],\\ d_{i}=\overset{n}{\underset{j=1}{\sum }}t_{D}^{ij},i=1,2,\ldots ,n.\\ W^{\alpha }=T_{D}^{\alpha }W \end{array}$$
Step 3 – Calculated the ultimate supermatrix W^*^.
  $$\begin{array}{l} W^{*}=\underset{g\to \infty }{\lim}{\left(W^{\alpha }\right)}^{g} \end{array}$$
Step 4 – Calculated the mixed weight as per the following formula:
  $$\begin{array}{l} Z=w+T\times w=\left(I+T\right)w \end{array}$$
  where, *Z* is the mixed weight and W is the comprehensive weight of secondary indicators.

### Data

In order to ensure the data quality and quantity requirements of the DANP method, we have done following efforts. In terms of data quality, since sample's appropriateness and richness is very important (93), this paper selects qualified experts based on three criteria. First, experts are experienced and have at least 15 years of research or work experience in food safety areas. Second, experts must have an academic professorship, food industrial manager, or a government food safety governor background, in order to possess a more comprehensive knowledge structure. This determines the diversity, representativeness and breadth of the expert group, and can give a comprehensive evaluation based on the comprehensive consideration of the views and interests of different stakeholders related to food fraud. Third, experts all must be from China.

It should be noted that the research method used in this paper does not require a high number of experts to participate in the evaluation. For example, when Chiu and Tzeng (94) and Shen and Tzeng (95) used DANP (DEMATEL-based ANP) to conduct the study, the number of experts participating in the evaluation was only eight. Thus, our study refers to the literature of Chiu and Tzeng (94), Shen and Tzeng (95), Chuang and Chen (96), and Huang et al. (36), and uses the average deviation rate (or what is referred to as “errors of gap ratio”) to determine the number of experts, which satisfies the number of participating experts in the evaluation process as required by the DANP method. In terms of data quantity, according to Chiu et al. (94), Huang et al. (36), Chuang and Chen (96) and Shen and Tzeng (95), this paper uses the average deviation rate to assess whether the expert size reaches theoretical saturation $\left(\frac{1}{n(n-1)}\sum_{i=1}^{n}\sum_{j=1}^{n}\frac{\left|a_{ij}^{p}-a_{ij}^{p-1}\right|}{a_{ij}^{p}}\times 100\%\right)$. *p* is the number of experts, $a_{ij}^{p}$ is the average effect of factor *i* on factor *j*, and *n* is the number of factors being affected. In this paper, a group of experts were invited to participate in the project, who come from China National Food Industry Association, China Agricultural University, Shandong Agricultural University, Jiangnan University, Jiangsu Academy of Agricultural Sciences and other institutions. Experts can express their opinions and discuss together before evaluating the relationship between the two factors. Since the opinions of the experts are expressed in terms of language rather than numerical values, when the evaluation results are finally collected, experts are required to score the pairwise relationship between the factors according to the corresponding integer values in Table 2.

**Table 2:** Conversion between linguistic variables and integer rank.

**Linguistic variable**	**Corresponding integer**
No (no influence)	0
VL (very low influence)	1
L (low influence)	2
H (high influence)	3
VH (very high influence)	4

Finally, regarding the theoretical saturation, we refer to Chuang and Chen (96) for our study. Using the average deviation rate (or “errors of gap ratio,” EGR) method, we calculated that the average deviation rate of the nine experts who participated in the evaluation was 4.25% <5% (see P24 of the revised paper). This indicates that we have more than 95% confidence that there is no significant difference between the results of 9 experts and 8 experts participating in the evaluation. According to Chuang and Chen (96), it is reasonable to assume that 9 experts are close to the theoretical saturation and meet the requirement of an appropriate number of experts.

## Results

By averaging the expert assessment results, we obtained the direct relationship average matrix *A*. By repeating the above step, we then obtained the initial direct relationship matrix *D* (Table 3), line sum and column sum (*r*_*i*_ and *c*_*i*_) of total influence matrix *T* and of each cluster and factor (Table 4), and the mixed weights of the clusters and factors. Finally, we performed normalized sorting of the mixed weights to compile (Table 5).

**Table 3:** Initial direct relationship matrix *D*.

	**C_**11**_**	**C_**12**_**	**C_**13**_**	**C_**21**_**	**C_**22**_**	**C_**31**_**	**C_**32**_**	**C_**33**_**	**C_**41**_**	**C_**42**_**	**C_**51**_**	**C_**52**_**
C_11_	0.00000	0.07609	0.08333	0.09058	0.07609	0.03261	0.02899	0.03623	0.03261	0.05435	0.07971	0.08696
C_12_	0.06884	0.00000	0.11957	0.08333	0.03623	0.07609	0.07609	0.05435	0.10145	0.06159	0.08696	0.07971
C_13_	0.07246	0.11594	0.00000	0.08696	0.05435	0.08696	0.08696	0.05072	0.10870	0.06522	0.08696	0.07609
C_21_	0.07246	0.07246	0.07246	0.00000	0.10145	0.10507	0.09783	0.07609	0.10145	0.07246	0.08696	0.06884
C_22_	0.09058	0.05797	0.05072	0.09420	0.00000	0.07609	0.06884	0.09058	0.07971	0.05435	0.09783	0.07246
C_31_	0.08696	0.09420	0.07246	0.10870	0.09420	0.00000	0.10145	0.08333	0.09783	0.06522	0.09420	0.10145
C_32_	0.06884	0.07971	0.07609	0.08333	0.07971	0.09783	0.00000	0.09058	0.09783	0.08333	0.08696	0.08333
C_33_	0.06884	0.08333	0.04710	0.07609	0.10145	0.08333	0.04348	0.00000	0.06522	0.06159	0.07246	0.04348
C_41_	0.10507	0.11232	0.08696	0.07609	0.03986	0.09783	0.09420	0.03261	0.00000	0.10145	0.07246	0.09420
C_42_	0.02899	0.07246	0.07246	0.06522	0.05072	0.09783	0.10145	0.05797	0.10145	0.00000	0.08696	0.09420
C_51_	0.08696	0.09058	0.06884	0.08333	0.09058	0.08333	0.06522	0.06159	0.06884	0.05797	0.00000	0.09420
C_52_	0.07246	0.09783	0.07609	0.09420	0.07246	0.08333	0.08696	0.06522	0.09783	0.05797	0.09783	0.00000

**Table 4:** Values of *r*_*i*_, *c*_*i*_, *r*_*i*_ + *c*_*i*_, and *r*_*i*_ − *c*_*i*_ for clusters and factors influencing enterprise food fraud.

**Cluster**	***r*_*i*_**	***c*_*i*_**	***r*_*i*_ − *c*_*i*_**	***r*_*i*_ + *c*_*i*_**	**Factor**	***r*_*i*_**	***c*_*i*_**	***r*_*i*_ − *c*_*i*_**	***r*_*i*_ + *c*_*i*_**
D_1_	2.53517	2.72529	−0.19012	5.26046	C_11_	5.11596	6.20740	−1.09144	11.32336
					C_12_	6.35550	7.08381	−0.72831	13.43931
					C_13_	6.67952	6.24157	0.43795	12.92110
D_2_	2.62924	2.59154	0.0377	5.22078	C_21_	6.91238	6.99331	−0.08093	13.90570
					C_22_	6.19436	5.94982	0.24455	12.14418
D_3_	2.77796	2.58851	0.18945	5.36647	C_31_	7.38820	6.83774	0.55046	14.22594
					C_32_	6.89975	6.40159	0.49816	13.30135
					C_33_	5.59856	5.26934	0.32922	10.86790
D_4_	2.73436	2.63741	0.09695	5.37177	C_41_	6.80847	7.07836	−0.26989	13.88683
					C_42_	6.31226	5.56394	0.74832	11.87619
D_5_	2.72901	2.86299	−0.13398	5.59200	C_51_	6.34861	7.02748	−0.67887	13.37610
					C_52_	6.73675	6.69597	0.04079	13.43272

**Table 5:** Normalized rank of mixed weights of clusters and factors influencing enterprise food fraud.

	**Influence weight**	**Rank**	**Criterion**	**Influence weight**	**Rank**
D_1_	0.23108	2	C_11_	0.06623	12
			C_12_	0.08112	10
			C_13_	0.08373	7
D_2_	0.17232	5	C_21_	0.09111	2
			C_22_	0.08121	9
D_3_	0.24903	1	C_31_	0.09245	1
			C_32_	0.08634	5
			C_33_	0.07024	11
D_4_	0.17293	4	C_41_	0.09057	3
			C_42_	0.08236	8
D_5_	0.17464	3	C_51_	0.08541	6
			C_52_	0.08922	4

## Discussion

Based on the calculation results obtained by the DANP method, this section identifies the interrelationships between Clusters and Factors that affect food counterfeiting and the intrinsic mechanisms that influence counterfeiting decisions of food companies, and identifies the key Clusters and key Factors from three aspects.

### Relationships Among Clusters and Factors That Influence Enterprise Food Fraud

**Figure 1:**
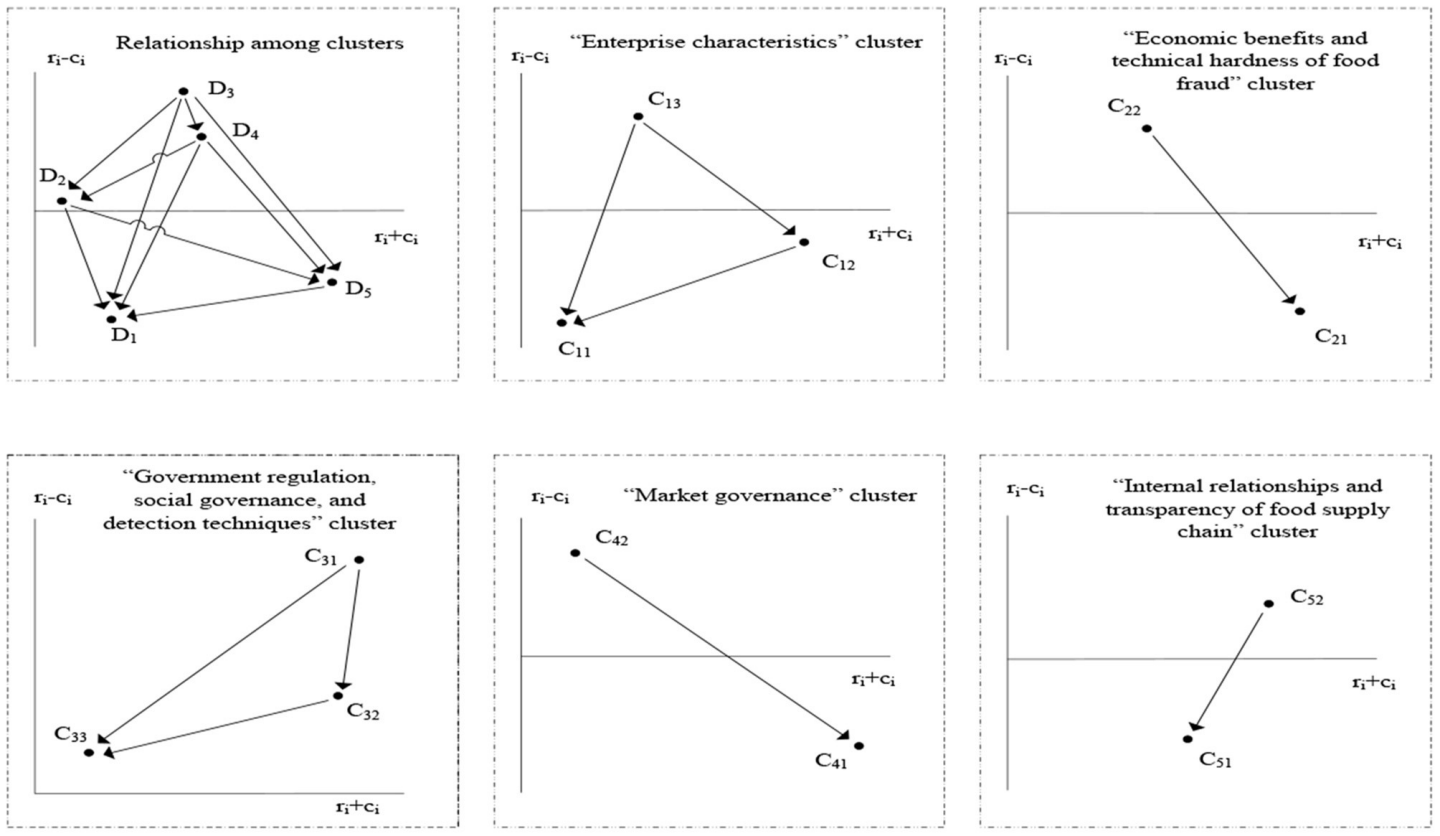
Influence relationship net map among clusters and factors.

The *r*_*i*_ − *c*_*i*_ and *r*_*i*_ + *c*_*i*_ values of each cluster and factor obtained from DEMATEL analysis are shown in Table 4. With reference to the plotting methods of Yang and Tzeng (97) and by use of the (*r*_*i*_ + *c*_*i*_, *r*_*i*_ − *c*_*i*_) dataset, we obtained the influential net relationship map (Figure 1).

Figure 1 depicts the direct relationships among five clusters that influence enterprise food fraud, i.e., enterprise characteristics (*D*_1_), economic benefits and technical hardness of food fraud (*D*_2_), government regulation, social governance, and detection techniques (*D*_3_), market governance (*D*_4_), and internal relationship and transparency of food supply chain (*D*_5_). The direct influence of cluster *D*_3_ on *D*_4_, *D*_2_, *D*_5_, and *D*_1_ can be expressed as *D*_3_ → {*D*_4_, *D*_2_, *D*_5_, *D*_1_}. Similarly, the direct influence of cluster *D*_4_ on *D*_2_, *D*_5_, and *D*_1_ can be expressed as *D*_4_ → {*D*_2_, *D*_5_, *D*_1_}; the direct influence of cluster *D*_2_ on *D*_5_ and *D*_1_ can be expressed as *D*_2_ → {*D*_5_,*D*_1_}; and the direct influence of cluster *D*_5_ on *D*_1_ can be expressed as *D*_5_ → {*D*_1_}.

Figure 1 also shows the direct influence relationship among factors within the same cluster. For example, cluster *D*_1_ encompasses three interrelated factors, i.e., enterprise scale (*C*_11_), business ethics (*C*_12_), and manager's awareness of social responsibilities (*C*_13_). The direct influence of *C*_13_ on *C*_12_ and *C*_11_ can be expressed as *C*_13_ → {*C*_12_,*C*_11_} and the direct influence of *C*_12_ on *C*_11_ can be expressed as *C*_12_ → {*C*_11_}. The direct influence relationship among factors within each of the other four clusters can be expressed in the same way as cluster *D*_1_.

### Intrinsic Mechanism of How Various Clusters and Factors Influence Enterprise Food Fraud

The *r*_*i*_ − *c*_*i*_ values in Table 4 were used to determine by what intrinsic mechanism the clusters and factors influence enterprise food fraud. Firstly, at the cluster level, *D*_3_, *D*_4_, and *D*_2_ were identified as cause clusters based on their positive *r*_*i*_ − *c*_*i*_ values, with each influencing other clusters in the system to a certain degree. In addition, *D*_1_ and *D*_5_ were identified as result clusters based on their negative *r*_*i*_ − *c*_*i*_ values, with both influenced significantly by other clusters in the system. Therefore, the five clusters interacted intrinsically, such that clusters *D*_3_, *D*_4_, and *D*_2_ directly and/or indirectly influenced clusters *D*_1_ and *D*_5_, and ultimately enterprise food fraud. This intrinsic mechanism can help us understand the causes of food fraud. In developed countries, the lack of detection technology is an important cause of food fraud (6). However, the Figure 1 shows that in China, the lack of government governance is highly related to insufficient supervision of social entities, but not for the reasons of governance approaches. This result might also apply to, and have implications for, other developing countries.

At the factor level, seven factors were identified as cause factors based on their positive *r*_*i*_ − *c*_*i*_ values, with each imposing significant influence on other factors in the system to varying degrees. These factors included consumption behavior on food market (*C*_42_), government regulatory capability and penalty intensity (*C*_31_), supervision by social forces (*C*_32_), manager's awareness of social responsibility (*C*_13_), technical hardness (*C*_22_), utility of detection techniques and methodologies (*C*_33_), and transparency of supply chain (*C*_52_). The other five factors were identified as result factors based on their negative *r*_*i*_ − *c*_*i*_ values, with each influenced significantly by other factors to varying degrees. These factors included enterprise scale (*C*_11_), business ethics (*C*_12_), constraints by downstream enterprises (*C*_51_), expected economic benefits (*C*_21_), and maturity of market reputation mechanism (*C*_41_). In summary, the factors interacted and influenced the fraudulent behavior of food enterprises intrinsically, with *C*_42_,*C*_31_, *C*_32_,*C*_13_,*C*_22_,*C*_33_, and *C*_52_ directly and/or indirectly influencing *C*_11_, *C*_12_, *C*_51_, *C*_21_, and *C*_41_, and ultimately enterprise food fraud. From a supply perspective, an in-depth understanding of the unethical behavior of companies pursuing profits in the supply chain can help us understand the food fraud behavior of companies (1). However, this intrinsic mechanism further reveals the particularity of the causes of Chinese food fraud from the perspective of demand. As in Table 4, *C*_42_′*sr*_*i*_ − *c*_*i*_ value is the largest, indicating that the consumption behavior of the food market, especially the food literacy of consumers, provides a market space for food fraud. This may also be an important reason why food fraud in rural China is more serious than in urban areas.

In addition to the above, another major advantage of the DANP method is that when a result factor emerges, the decision-maker can determine what has caused the issue by examining the cause factors. Take the internal relationships and transparency of the food supply chain (*D*_5_) cluster as an example. Table 4 shows that constraints by downstream enterprises (*C*_51_) was the only result factor in this cluster, whereas transparency of supply chain (*C*_52_) was the cause factor. Loose constraints on upstream enterprises by downstream enterprises on the supply chain may be due to inadequacy of supply chain transparency. Similarly, low manager awareness of social responsibilities may be due to small scale or poor business ethics of the enterprise. High expected economic benefits from food fraud may be due to the low technical hardness of fraud. These inferences conform to what occur in the real world and may provide essential references for the government in stipulating and enforcing relevant policies.

### How to Identify the Clusters and Factors Influencing Enterprise Food Fraud

Based on the internal relationships among clusters and factors and the intrinsic mechanism of how they influence enterprise food fraud, we used the mixed weights in Table 5 to further identify the key clusters and factors that influence enterprise food fraud.

Results demonstrated that the government regulation, social governance, and detection techniques (*D*_3_) cluster had an influence weight of 0.24903, and thus was a key cluster ranking first among the five clusters, as also seen in Figure 1. Furthermore, *D*_3_ had the maximum *r*_*i*_ − *c*_*i*_ value, which did not differ significantly from that of *D*_5_. This implies that, as a key cluster, *D*_3_ significantly influenced the other clusters and played a dominant role in the system. Therefore, based on the mixed weights, the DANP results were consistent with those obtained using DEMATEL. The results showed that the relationships between dimensions and real-world considerations are more significant than any single dimension. This also reveals the importance of establishing a system of social co-governance (implemented by improving all dimensions but not any single one) that is jointly supervised by the government and social entities in China.

Secondly, factors with a mixed weight > 0.09 in Table 5 were identified as key factors that influence the food fraud behavior of enterprises. Government regulation and penalty intensity (*C*_31_) was deemed a key factor based on its first-ranked mixed weight of 0.09245. This is consistent with the conclusions of Lord et al. (47) and Kendall et al. (54). The expected economic benefits (*C*_21_) and maturity of market reputation mechanism (*C*_41_) were also deemed as key factors with mixed weights of 0.09111 and 0.09057, ranking second and third, respectively. These findings are supported by Charlebois et al. (68). Transparency of supply chain (*C*_52_) was also determined to be a key factor, with a mixed weight of 0.08922 (close to 0.09), ranking fourth in the system. This result is supported by Bitzios et al. (70).

The key factors identified above are consistent with previous studies, thus providing preliminary proof that the DANP method is applicable and the conclusions of the study are reliable. Furthermore, to verify the applicability of the DANP method, we compared the key factors identified by DEMATEL and DANP analyses, which showed consistent conclusions. Previous studies have generally identified key factors by the magnitude of the *r*_*i*_+*c*_*i*_ values obtained using DEMATEL (55). As seen in Table 4, the first four key factors ranked by the DEMATEL *r*_*i*_ + *c*_*i*_ values were government regulation and penalty intensity, expected economic benefits from fraud, maturity of market reputation mechanism, and transparency of supply chain. These results agree with the conclusions obtained using the mixed weight magnitudes from DANP (Table 5). Therefore, it is reasonable to believe that the four key factors proposed by this paper are accurate. Thus, we found the DANP method to be applicable in the identification of key factors that influence enterprise food fraud behavior.

In addition to the four key factors above, six other factors, namely supervision by social forces (*C*_32_), constraints by downstream enterprises (*C*_51_), manager's awareness of social responsibility (*C*_13_), consumption behavior on food market (*C*_42_), technical hardness of food fraud (*C*_22_), and enterprise business ethics (*C*_12_), had mid-rank mixed weights ranging from 0.8 to 0.9, and were thus deemed to be secondary key factors. Two further factors, namely utility of detecting techniques and methodologies (*C*_33_) and enterprise scale (*C*_11_), ranked last in the system and were therefore deemed to be non-key factors. As seen from most food safety incidents in China, food fraud is primarily uncovered by simple detection. Thus, the utility of detection methodologies is not directly related to enterprise food fraud. Furthermore, although it is generally recognized that enterprise scale can influence fraudulent behavior (28, 55), this was not supported in the current study. It is possible that food fraud occurs frequently in China and enterprises can commit food fraud regardless of enterprise scale. Therefore, food fraud may not be necessarily associated with enterprise scale.

## Policy Implications and Conclusions

### Policy Implications

In a complex system encompassing multiple stakeholders, we found that enterprise food fraud was subject to joint influences by multiple clusters. Government regulation, social governance, and detection techniques was the key cluster. Furthermore, government regulatory capability and penalty intensity, expected economic benefits from fraud, maturity of market reputation mechanism, and transparency of food supply chain were the four key factors. We further determined the intrinsic mechanism of fraudulent behaviors of food enterprises and demonstrated that the DANP method is effective at identifying key clusters and factors that influence enterprise food fraud.

The current research was based on participation of a group of experts and was conducted within the context in China's food systems. One common attribute is that all of the experts have deep care and understanding of policy making regarding food fraud. Thus, the results could have profound policy implications from the social co-governance perspective for China and similar economies. First, Fraudulent behavior depends not only on expected economic benefits but also on expected cost (i.e., probability of getting caught and the penalty if they are caught cheating). Among them, the probability of being caught is determined by factors such as the effectiveness of detection techniques and methods (i.e., utility of detection Techniques and methodologies), and the supervision of social forces. The punishment after being caught is determined by factors such as Government Penalty Intensity and Maturity of Market Reputation Mechanism. Due to the major attractive effect of expected economic benefits of committing food frauds for enterprises, the government should be increased penalty of getting caught, so that the economic costs of food fraud are increased to a level sufficient to change the psychological expectation for economic return of food fraud. From a social co-governance perspective, not only the government should exercise such a penalty system. Business partners (e.g., suppliers or buyers), for example, could exercise such penalty method by contract; while end consumers could exercise such penalty by collective actions of refusing purchases (98, 99). In addition, in addition to strengthening supervision and sampling and improving the level of detection technology, it is also necessary to actively promote internal employees to provide food fraud clues.

Second, a regulation mechanism based on individual person's and an enterprise's life-long, public credit should be established. Food enterprises should be rated by credit levels and regulation should differ for the different levels, including punitive measures and close-out mechanisms against credit-losing enterprises. With such system, all stakeholders could see the credit and collectively perceive the credibility of a food enterprise.

Third, priority should be given to criminal liabilities. In parallel with behavioral and property punishments, confinement should be stressed, i.e., administrative detention of the responsible persons. By eliminating no or weak enforcement and limited economic penalties in substitution for stronger criminal liabilities, a lasting system-based mechanism and legislative environment will be established to ensure that food enterprises are unable to or do not wish to commit food fraud.

Fourth, the market reputation mechanism should be leveraged to control food fraud by disclosing food fraud information in a widespread manner through public media.

Fifth, a food traceability system should be established, and the food supply chain should have due transparency. Government authorities should establish and popularize food traceability systems and ensure food enterprises maintain continuous records to create reliable information flow along the supply chain, thus allowing food production processes and destinations to be monitored, food fraud to be identified by tracking, and recall to be ordered when necessary. These measures will, in turn, encourage food enterprises to maintain compliance in business operation.

Sixth, although the food fraud vulnerability assessment tools are still in their infancy, its full impact remains to be seen. However, over time, food fraud vulnerability assessment tools can be used to ensure the food supply chain. Play an active role in integrity (100). China should also actively promote and encourage companies to implement food fraud vulnerability assessments. This is also an important part of social co-governance.

### Conclusions

This paper adopts the DANP approach to make up for the deficiencies of existing studies that do not examine the key factors (cluster) and the interrelationships between factors (cluster) that influence food enterprises' food fraud decisions from the perspective of social co-governance and business decision making, thus contributing to an in-depth understanding of the causes of food fraud by food enterprises and to the formulation of targeted. The study contributes to the understanding of the causes of food fraud in food companies, and to the formulation of targeted measures to change the decisions of food companies and reduce food fraud at source.

Theoretically and practically, a social co-governance perspective extends the scope of governance to a multiple-agent level. That is, not just the producer enterprise is the focus of fraud prevention, but all stakeholders become the ones being governed by all of other social actors. The system design thinking of a food fraud governance should be a dominant logic to cover all government needs, whether which social actor is the one who is governing or governed.

What needs to be explained is the government regulatory capability and penalty intensity. Government has two instruments to control food fraud: (1) certification and (2) monitoring and enforcement system (31). The major reason for not discussing about the certification in the scope of the present study are: First, this paper is based on China's information. In China, the government's approach to countering food fraud is mainly government supervision and punishment, not certification. Second, in China, the government still needs to continuously improve the average product quality level in the market. When the government wants to increase the average product quality in the market while combating food adulteration, strict monitoring and enforcement is more effective than increasing certification costs (31). Therefore, this article will not discuss certification issues for the time being.

Additionally, in China, both legal food producers who have obtained food production licenses and a large number of illegal food producers who have not obtained licenses (such as illegal workshops) may engage in food fraud. The enterprise in this article refers to a legal food producer who has obtained a food production license. At the same time, we believe that the research conclusions are also applicable to illegal food producers who have not obtained a license to a certain extent.

## Data Availability Statement

The raw data supporting the conclusions of this article will be made available by the authors, without undue reservation.

## Author Contributions

All authors listed have made a substantial, direct, and intellectual contribution to the work and approved it for publication.

## Funding

This study was partially supported by the National Social Science Fund of China: Research on social co-governance of food safety risks and cross-border cooperative governance mechanism (20&ZD117).

## Conflict of Interest

The authors declare that the research was conducted in the absence of any commercial or financial relationships that could be construed as a potential conflict of interest.

## Publisher's Note

All claims expressed in this article are solely those of the authors and do not necessarily represent those of their affiliated organizations, or those of the publisher, the editors and the reviewers. Any product that may be evaluated in this article, or claim that may be made by its manufacturer, is not guaranteed or endorsed by the publisher.
